# Role of *Helicobacter pylori cagA* EPIYA motif and *vacA* genotypes for the development of gastrointestinal diseases in Southeast Asian countries: a meta-analysis

**DOI:** 10.1186/1471-2334-12-223

**Published:** 2012-09-21

**Authors:** Shu Sahara, Mitsushige Sugimoto, Ratha-Korn Vilaichone, Varocha Mahachai, Hiroaki Miyajima, Takahisa Furuta, Yoshio Yamaoka

**Affiliations:** 1 First Department of Medicine, Hamamatsu University School of Medicine, Hamamatsu, Japan; 2 Gastroenterology Unit, Department of Medicine, Thammasat University Hospital, Pathumthani, Thailand; 3 Gastroenterology Unit, Department of Medicine, Chulalongkorn University Hospital, Bangkok, Thailand; 4 Center for Clinical Research, Hamamatsu University School of Medicine, Hamamatsu, Japan; 5 Department of Environmental and Preventive Medicine, Faculty of Medicine, Oita University, Yufu, Japan

**Keywords:** *Helicobacter pylori*, *cagA*, *vacA*, EPIYA, Gastric cancer, Peptic ulcer disease, Southeast Asian

## Abstract

**Background:**

Infection with *cagA*-positive, *cagA* EPIYA motif ABD type, and *vacA* s1, m1, and i1 genotype strains of *Helicobacter pylori* is associated with an exacerbated inflammatory response and increased risk of gastroduodenal diseases. However, it is unclear whether the prevalence and virulence factor genotypes found in Southeast Asia are similar to those in Western countries. Here, we examined the *cagA* status and prevalence of *cagA* EPIYA motifs and *vacA* genotypes among *H. pylori* strains found in Southeast Asia and examined their association with gastroduodenal disease.

**Methods:**

To determine the *cagA* status, *cagA* EPIYA motifs, and *vacA* genotypes of *H. pylori*, we conducted meta-analyses of 13 previous reports for 1,281 *H. pylori* strains detected from several Southeast Asian countries.

**Results:**

The respective frequencies of *cagA*-positive and *vacA* s1, m1, and i1 genotypes among examined subjects were 93% (1,056/1,133), 98% (1,010/1,033), 58% (581/1,009), and 96% (248/259), respectively. Stratification showed significant variation in the frequencies of *cagA* status and *vacA* genotypes among countries and the individual races residing within each respective country. The frequency of the *vacA* m-region genotype in patients infected with East Asian-type strains differed significantly between the northern and southern areas of Vietnam (*p* < 0.001). Infection with *vacA* m1 type or *cagA*-positive strains was associated with an increased risk of peptic ulcer disease (odds ratio: 1.46, 95%CI: 1.01-2.12, p = 0.046 and 2.83, 1.50-5.34, *p* = 0.001, respectively) in the examined Southeast Asian populations.

**Conclusions:**

Both Western- and East Asian-type strains of *H. pylori* are found in Southeast Asia and are predominantly *cagA*-positive and *vacA* s1 type. In Southeast Asia, patients infected with *vacA* m1 type or *cagA*-positive strains have an increased risk of peptic ulcer disease. Thus, testing for this genotype and the presence of *cagA* may have clinical usefulness.

## Background

*Helicobacter pylori* infection is closely associated with the development of peptic ulcer disease and gastric cancer [1-4]. *H. pylori* colonizes the gastric mucosa of 50% of the world’s population, with infection levels exceeding 70% in developing areas, such as Latin America and Africa [5-10]. Several Southeast Asian countries also have a high prevalence of *H. pylori* infection, particularly Thailand, where the *H. pylori* infection rate ranges from 54.1%-76.1% [11,12]. Therefore, developing countries, including those located in Southeast Asia, are considered to have a higher incidence of *H. pylori*-related diseases. However, the estimated age-standardized incidence rate of gastric cancer in Southeast Asian countries (10.2 and 4.7/100,000 in men and women, respectively) is lower than those in East Asia (e.g., 124.63/100,000 in Japan and 48.25/100,000 in Korea) and South America (e.g., 33.22/100,000 in Colombia) ( http://globocan.iarc.fr/). This phenomenon, which is characterized by high levels of *H. pylori* infection, but a low incidence of gastric cancer, is referred to as the “Asian paradox” [13,14].

Recently, numerous studies have examined the relationships between *H. pylori* virulence factors and outer membrane proteins with gastric mucosal inflammation and gastroduodenal disease development [9,10,15,16]. The *cag* PAI including of *cagA* encodes a putative type IV secretion system, which transfers a variety of multimolecular complexes, such as CagA, and across the bacterial membrane to the extracellular space or into other attached host cells [17,18]. *H. pylori cagA* plays important roles in gastric mucosal inflammation and injury in relation to activated inflammatory cells infiltration [19,20]. Activated neutrophils and mononuclear cells infiltrating into gastric mucosa with *H. pylori* infection produce several pro-inflammatory cytokines (e.g., IL-1β, IL-6, IL-8 and TNF-α) and anti-inflammatory cytokines (e.g., IL-4 and IL-10). Greater than 90% of *H. pylori* strains isolated from East Asian populations carries the *cagA* gene [7,21]. In contrast, 40% of strains isolated in Western countries are *cagA*-negative [7,21]. Moreover, as tyrosine phosphorylation of CagA occurs at EPIYA sites consisting of five amino acid residues, the structure and role of the *cagA* EPIYA motif in *cagA* gene have been investigated [22-24]. The EPIYA motif exhibits genetic variation that occurs in four distinct segments, the EPIYA-A, -B, -C, and –D segments [22-24]. The representative CagA of Western *H. pylori* strains possesses a single EPIYA-A and EPIYA-B segment, followed by a 34-amino-acid EPIYA-C segment (EPIYA-ABC type). The C-terminal regions of East Asian and Western CagA are characterized by the presence of EPIYA-ABD and -ABC segments, respectively [22-24]. In addition, we [7] reported that *H. pylori* strains isolated from East Asian and Western countries could be completely distinguished by polymerase chain reaction-based *cagA* 5’ and 3’ region genotyping, and named the East Asian type *H. pylori* strains as *cagA* 1a type and the Western type *H. pylori* strains as *cagA* 2a type. *CagA* 1a type is correspondent roughly to Western-type *cagA* (EPIYA-ABC) genotype and 2a type to East Asian-type *cagA* (EPIYA-ABD) genotype. However, it is unclear whether the cagA EPIYA motif is associated with the development of gastrointestinal disease in Southeast Asian populations.

Gastric epithelial cell injury associated with *H. pylori* infections is caused by a vacuolating cytotoxin encoded by the *H. pylori vacA* gene. The *vacA* signal (s) region encodes the signal peptide and N-terminus of processed *vacA* toxin. In *H. pylori*, the *vacA* s1 genotype is associated with fully active toxin, but type 2 genotype strains produce *vacA* with a short N-terminal extension that blocks vacuole formation [25]. The *vacA* middle (m) region encodes part of the 55-KDa subunit located at the C-terminus and has two genotypes (m1 and m2); the former causes stronger vacuolating activities than the latter [25]. Recently, a third polymorphic determinant of vacuolating activity located between the s- and m- regions was identified and termed the intermediate (i) region [26]. In general, the *vacA* s1, m1, and i1 genotypes of *H. pylori* are associated with an increased risk of disease due to the enhanced production of toxin with markedly higher vacuolating activity than that of *vacA* s2, m2, and i2 genotype strains, which are rarely associated with peptic ulcer disease and gastric cancer [7,8,15,25,27-30]. However, it is unclear whether this association is observed in *H. pylori* strains commonly found in Southeast Asian populations.

To date, 13 studies have investigated the *cagA* status and genotyping of *cagA* EPIYA motifs and *vacA* s, m, and i-regions in *H. pylori* strains found among Southeast Asian populations (Table 1) [7,31-42]. However, due to the small sample size in each report, it remains unclear whether *cagA*-positive strains and the EPIYA motif and *vacA* genotypes are associated with an increased risk for gastrointestinal disease in Southeast Asian populations [7,31-42]. Therefore, the present study was designed to elucidate the relationship between *H. pylori* virulence-factor genotypes and *H. pylori-*related disease susceptibility in *H. pylori*-infected patients living in Southeast Asia.

**Table 1:** **Reported ***cagA ***genotypes and *vacA *s, m, and i regions genotypes and ***cagA ***status in studies used in the present meta-analysis**

					***cagA *genotype**					***vacA *genotype**					
**Area/country**	**Authors**	**Year**	Patients (n)^*^	***cagA***	**ABC**	**ABCC/ABCCC**	**ABD**	**2a**	**1a**	**s1 (n/%)**	**s2 (n/%)**	**m1 (n/%)**	**m2 (n/%)**	**i1 (n/%)**	**i2 (n/%)**
Vietnam	Yamaoka Y [7]	2002	25 ^S^	24 (96)	NA	NA	NA	0 (0)	24 (100)	25 (100)	0 (0)	14 (56)	11 (44)	NA	NA
Vietnam	Uchida T [31]	2009	103 ^S^	98 (95)	4 (4)	0 (0)	94 (96)	NA	NA	103 (100)	1 (1)	44 (45)	54 (55)	NA	NA
Vietnam	Truong BX [32]]	2009	22 ^S^	22 (100)	1 (5)	0 (0)	21 (95)	NA	NA	NA	NA	NA	NA	NA	NA
Vietnam	Nguyen TL [33]	2010	100	95 (95)	NA	NA	NA	NA	NA	100 (100)	0 (0)	48 (48)	52 (52)	94 (94)	6 (6)
Thailand	Yamaoka Y [7]	2002	8 ^S^	8 (100)	NA	NA	NA	4 (50)	4 (50)	8 (100)	0 (0)	6 (75)	2 (25)	NA	NA
Thailand	Vilaichone RK [34]	2004	98 ^S^	98 (100)	NA	NA	NA	50 (51)	48 (49)	98 (100)	0 (0)	71 (72)	27 (28)	NA	NA
Thailand	Linpisarn S [35]	2007	135	119 (88)	NA	NA	NA	NA	NA	132 (100)	0 (0)	73 (63)	42 (37)	NA	NA
Thailand	Chomvarin C [36]	2008	112 ^S^	110 (98)	NA	NA	NA	NA	NA	112 (100)	0 (0)	65 (58)	47 (42)	NA	NA
Malaysia	Tan HJ [37]	2005	127 ^S^	107 (84)	NA	NA	NA	NA	NA	117 (92)	10 (8)	81 (64)	46 (36)	NA	NA
Malaysia	Tan HJ [38]	2006	73 ^S^	58 (79)	NA	NA	NA	NA	NA	65 (89)	8 (11)	46 (63)	27 (37)	NA	NA
Malaysia	Mohamed R [39]	2009	93	NA	23 (22)	13 (12)	70 (66)	NA	NA	NA	NA	NA	NA	NA	NA
Malaysia	Schmidt HMA [40]	2009	126 ^S^	NA	34 (28)	19 (16)	68 (56)	NA	NA	NA	NA	NA	NA	NA	NA
Malaysia	Schmidt HMA [41]	2010	159 ^S^	159 (100)	71 (45)	0 (0)	88 (55)	NA	NA	155 (97)	4 (3)	94 (59)	65 (41)	154 (97)	5 (3)
Singapore	Zheng PY [42]	2000	108 ^S^	95 (88)	NA	NA	NA	NA	NA	103 (99)	1 (1)	39 (38)	65(62)	NA	NA

‘NA’ (not available) indicates a lack of data associating *cagA* status, EPIYA motif genotype, and *vacA* s-, m-, and i-region genotypes.

The uppercase ‘s’ indicates the use of single colonies and the remaining studies refer to the use of gastric biopsy samples, paraffin-embedded biopsy samples, and pools of cultured colonies.

## Methods

### Study selection

A literature search was performed using the PubMed databases for articles written in English and published before June 2011. The following search words were used: 1) *cagA* or EPIYA, 2) *vacA* or vacuolating cytotoxin, 3) *pylori* or *Helicobacter*, and 4) genotype. We did not include abstracts or unpublished articles. We conducted a combined analysis to determine the prevalence of *cagA*-positive strains, *cagA* EPIYA motif genotypes, and *vacA* genotypes in *H. pylori* strains found in Southeast Asia and their association with gastrointestinal diseases.

### Inclusion Criteria

The following criteria were applied to select published case–control studies examining the relationship between *cagA* EPIYA motif genotype or *vacA* genotype and clinical outcomes in adult populations infected with *H. pylori* isolated in four Southeast Asian countries (Vietnam, Thailand, Singapore, and Malaysia): the presence and genotypes of the *cagA* EPIYA motif (ABC, ABCC, and ABD genotypes) and *vacA* (*vacA* s-, m-, and i- regions) were examined by polymerase chain reaction (PCR) and original articles published in English. The references cited in these manuscripts were also screened using the same inclusion criteria. When it appeared that the same subjects were included in multiple reports, only the earliest article was selected.

### Data analysis

As several studies did not measure all parameters simultaneously (*cagA* status and *vacA* s-, m-, and i-region genotypes), the patient and strain numbers (*H. pylori* genotype number) did not match in the following analyses.

Statistical differences in the prevalence of *cagA* status, EPIYA motif genotype, and *vacA* genotype among the individual countries and ethnic groups were determined by the chi-squared test and Fisher's exact test. The effects of *cagA* status, EPIYA motif genotype, and *vacA* genotypes on the risk of gastric cancer and peptic ulcer disease were expressed as odds ratios (ORs) with 95% confidence intervals (CIs) with reference to non-ulcer dyspepsia (NUD) subjects infected with *H. pylori*. NUD was defined as endoscopical gastritis with no peptic ulcer disease or gastric cancer. In this study, patients with ‘NUD’ were regarded as the control group. All *p* values were two-sided, and *p* values <0.05 were considered statistically significant. Calculations were carried out using the statistical software StatView 5.0 (SAS Institute, Cary, NC, USA). Meta-analyses were performed using Comprehensive Meta-Analysis software (version 2, Biostat, Englewood, NJ).

## Results

### Included studies

Seventeen studies investigating Southeast Asian populations were identified using our search criteria (Table 1). Notably, a report from Yamaoka et al. [7] included two populations: Vietnamese and Thai. Van Doorn et al. [43] included strains isolated from Southeast Asia, but did not report the country of origin. Studies by Ho et al. [44] and Vivatvakin et al. [45] did not provide detailed *vacA* genotype information, and a report from Yamaoka Y et al. [46] investigated Vietnamese living in Houston, Texas. Finally, a total of 13 studies with a combined 1,281 patients (4 reports for Vietnam, 4 from Thailand, 5 from Malaysia, and 1 from Singapore) were included in the systematic analyses (Tables 1 and 2).

**Table 2:** **Summary of *cagA *status*,*** EPIYA **motif genotype, and *vacA *s-, m- and i-region genotypes in different Southeast Asian countries**

				***cagA *genotype**		***vacA *genotype**												
	**Paper (n)**	**Patient (n)**	***cagA *(n/%)**	**Western EPIYA-ABC**	**East-Asian EPIYA-ABD**	**s1 (n/%)**	**s1a (n/%)**	**s1b (n/%)**	**s1c (n/%)**	**s2 (n/%)**	**m1 (n/%)**	**m2 (n/%)**	**s1m1 (n/%)**	**s1m2 (n/%)**	**s2m1 (n/%)**	**s2m2 (n/%)**	**i1 (n/%)**	**i2 (n/%)**
Vietnam	4	250	239 (96)	5 (3)	139 (97)	228 (100)	NA	NA	NA	1 (0)	106 (48)	117 (52)	106 (48)	117 (52)	0 (0)	0 (0)	94 (94)	6 (6)
Thailand	4	345	327 (95)	50 (51)	48* (49)	342 (100)	107 (62)	0 (0)	66 (38)	0 (0)	215* (65)	116 (35)	215* (65)	116 (35)	0 (0)	0 (0)	NA	NA
Malaysia	5	578	324 (90)	160 (43)	215* (57)	337 (94)	33 (51)	4 (6)	28 (43)	22 (6)	221* (62)	138 (38)	221* (62)	124 (35)	0 (0)	14 (4)	154 (97)	5 (3)
Singapore	1	108	95 (88)	NA	NA	103 (99)	NA	NA	NA	1 (1)	39 (38)	65 (63)	39 (38)	64 (62)	0 (0)	1 (1)	NA	NA
**Total**	13	1281	985 (93)	215 (35)	402 (65)	1010 (98)	140 (59)	4 (2)	94 (39)	24 (2)	581 (58)	436 (42)	581 (58)	421 (42)	0 (0)	15 (1)	248 (96)	11 (4)

‘NA’ (not available) indicates a lack of no data associating *cagA* status, EPIYA motif genotype, and *vacA* s-, m-, and i-region genotypes. As a report from Yamaoka Y, et al. included two populations: Vietnamese and Thai [7], the number of studies was reduced by one.

*: *p* < 0.05 (significant differences among different countries, when compared with Vietnam.) by use of chi-square test.

### Prevalence of *cagA* and *vacA* s, m, and i-region genotypes

**Figure 1:**
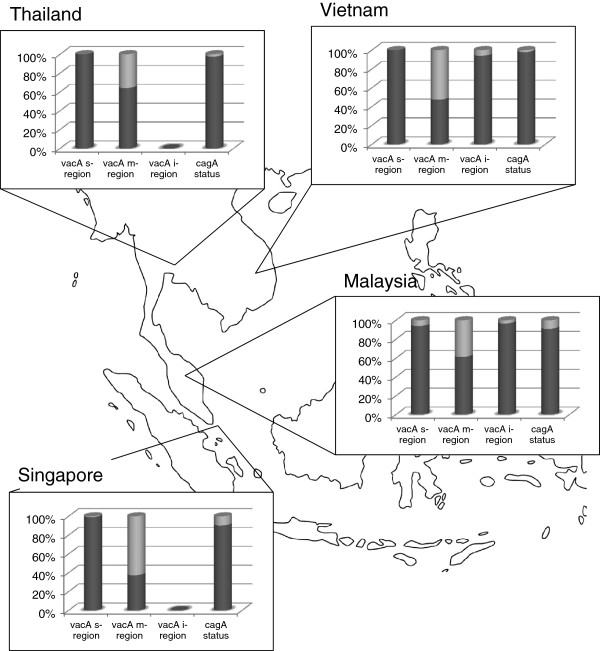
**Proportion of *vacA *s, m, and i genotypes and *cagA *status among *H. pylori *strains isolated from different Southeast Asia populations.** The frequencies of *vacA* m region genotype, but not *vacA* s and i region genotypes or *cagA* status, significantly differed among the examined Southeast Asia countries

In *H. pylori* strains isolated from Southeast Asia, the respective frequencies of *vacA* s1, m1, and i1 genotypes among the examined subjects were 98% (1,010/1,033), 58% (581/1,009), and 96% (248/259). The prevalence of the *vacA* m-region and combined *vacA* s/m genotypes differed significantly among isolates from the various Southeast Asian countries (*p* < 0.001) (Table 2 and Figure 1). However, no significant difference between the prevalence of *vacA* s-region genotypes was detected. The frequency of the *vacA* s2m1 combination genotype was close to 0%, which was in agreement with most previous studies examining other geographical regions [9,25]. Interestingly, the frequency of sub-genotypes of *vacA* s1, s1a and s1c, which are reported to be specific for East Asian *H. pylori* strains [7], were similar in Thailand and Malaysia (Table 2). Reports from Nguyen TL, et al. [33] and Schmidt HMA, et al. [41] investigated *vacA* i-region genotypes; however, the correlation with *vacA* i-region genotypes at the population level was unclear.

Of the 13 included studies, 93% of *H. pylori* strains were *cagA* positive. The strains were divided into the following two groups: East Asian strains, including the EPIYA-ABD genotype [22] and 1a type according to PCR-based *cagA* 5’ and 3’ region genotyping [7], and Western-type strains, including EPIYA-ABC and -ABCC genotypes and 2a type. The prevalence of East Asian- and Western-strain type *cagA* was 65% and 35%, respectively. In the Vietnam population, 97% (139/144) of strains was East-Asian strain type *cagA,* whereas *H. pylori* strains in Thailand and Malaysia consisted of approximately half of East Asian- and Western-type strains (Table 2).

Three studies compared *H. pylori* strains isolated from the northern (Hanoi) and southern parts (Ho Chi Minh) of Vietnam [31-33] (Tables 1 and 3). Although the prevalence of the *vacA* s1 genotype was identical between both areas (100%), the *vacA* m1 genotype was found at a significantly higher frequency in strains isolated from the north (58%; 60/104) than from strains detected in the south (34%, 32/94; *p* = 0.001). Therefore, the virulence of *H. pylori* in Vietnam may differ between the northern and southern areas of the country, despite having the same native population.

**Table 3:** **Differences in the distribution of *cagA *and *vacA *genotypes among different races residing within the same country**

				***cagA *genotype**		***vacA *genotype**												
	**Area/Race (n)**	**Patient (n)**	***cagA *(n/%)**	**Western EPIYA-ABC**	**East-Asian EPIYA-ABD**	**s1 (n/%)**	**s1a (n/%)**	**s1b (n/%)**	**s1c (n/%)**	**s2 (n/%)**	**m1 (n/%)**	**m2 (n/%)**	**s1m1 (n/%)**	**s1m2 (n/%)**	**s2m1 (n/%)**	**s2m2 (n/%)**	**i1 (n/%)**	**i2 (n/%)**
Vietnam	Hanoi (North)	107	103 (96)	1 (2)	51 (98)	107 (100)	NA	NA	NA	1 (1)	60* (58)	44 (42)	60* (58)	44 (42)	0 (0)	0 (0)	50 (94)	3 (6)
	Ho Chi Minh (South)	118	90 (94)	3 (7)	43 (93)	96 (100)	NA	NA	NA	0 (0)	32 (34)	62 (66)	32 (34)	62 (66)	0 (0)	0 (0)	44 (94)	3 (6)
Thailand	Thai	38	38 (100)	31^#^ (82)	7 (18)	38 (100)	31 (82)	0 (0)	7^#^ (18)	0 (0)	35^#^ (92)	3 (8)	35 (92)	3 (8)	0 (0)	0 (0)	NA	NA
	Thai-Chinese	40	40 (100)	16 (42)	24 (60)	40 (100)	17 (43)	0 (0)	23 (58)	0 (0)	28 (70)	12 (30)	28 (70)	12 (30)	0 (0)	0 (0)	NA	NA
	Chinese	20	20 (100)	3 (15)	17 (85)	20 (100)	5 (25)	0 (0)	15 (75)	0 (0)	8 (40)	12 (60)	8 (40)	12 (60)	0 (0)	0 (0)	NA	NA
Malaysia	Chinese	286	141 (95)	29** (13)	186 (87)	149 (100)	NA	NA	NA	0 (0)	82 (55)	67 (45)	82 (55)	67 (45)	0 (0)	0 (0)	90 (100)	0 (0)
	Indian	144	82 (94)	93 (89)	11 (11)	78 (90)	27 (82)	4 (12)	2 (6)	9 (10)	61 (70)	26 (30)	61 (70)	17 (20)	0 (0)	9 (10)	44 (90)	5 (10)
	Malay	75	43 (86)	25 (56)	19 (43)	45 (90)	23 (92)	0 (0)	2 (8)	5 (10)	32 (64)	18 (36)	32 (64)	13 (26)	0 (0)	5 (10)	22 (100)	0 (0)

‘NA’ (not available) indicates a lack of data associating *cagA* status, EPIYA motif genotype, and *vacA* s-, m-, and i-region genotypes.

*: *p* < 0.05 (significant differences between Northern and Southern parts in Vietnam) by use of chi-square test, #: *p* < 0.05 (significant differences among Thai, Thai-Chinese, and Chinese in Thailand) by use of chi-square test, **: *p* < 0.05 (significant differences among Chinese, Indian, and Malay in Malaysia) by use of chi-square test.

Thailand and Malaysia consist of individuals from neighboring populations. In Thailand, although the prevalence of the *vacA* m1 genotype was higher (64%) than that of the m2 genotype (36%), *vacA* m-region genotypes differed among Thai, Thai-Chinese, and Chinese populations. The prevalence of the *vacA* m1 genotype among native Thai, who represent the majority of the population (approximately 75%), was 92%, which was significantly higher than that in Chinese (40%, *p* < 0.001) (Table 3). In the analysis of the *vacA* s1 subtype, most strains isolated from Chinese patients were the s1c subtype (75%), which was significantly higher than that found in Thai and Thai-Chinese patients (*p* < 0.001). Moreover, the *cagA* EPIYA motif genotype significantly differed among the examined geographic populations (*p* < 0.001). Although East Asian-type *cagA* was predominant in Chinese living in Thailand, Western-type *cagA* was predominant in Thai patients, suggesting that most native Thai living in Thailand are infected with Western-type *H. pylori*. Therefore, this observation suggests that although *H. pylori* infections in Thai are predominantly due to *vacA* s1m1 type strains, which have high virulence, the strains possess Western-type *cagA*, which is associated with weak virulence compared with East Asian strains [47].

In Malaysia, the prevalence of *vacA* m1 genotypes was similar among Malay (64%), Chinese (55%), and Indian (70%) patients (p = 0.065) (Table 3). Most strains isolated from Indian and Malay patients were of the s1a subtype (82% and 92%, respectively). In the analysis of *cagA* EPIYA genotype, Chinese patients were predominantly infected with East Asian-type *cagA* (EPIYA-ABD)*,* whereas Indian patients were mostly infected with Western-type *cagA* (EPIYA-ABC). The incidence of Western-type *cagA* was slightly higher in Malay patients, who formed the majority of the population. The examination of strains isolated from different ethnic groups provided an opportunity to examine the hypothesis that ethnic groups tend to retain their ancestral *H. pylori* genotype after migration [7,46]. Intermarriage among ethnic Thai and Chinese in Thailand also allowed examination of the transmission pathways in relation to the ethnic status of the parents. When the mother was Chinese, 84% of the offspring possessed *H. pylori* with an East Asian genotype. By contrast, when mother was Thai and father Chinese, only 29% of strains among offspring were of East Asian types. This finding is consistent with the notion that the primary caretaker of the children is the most likely source of infection. Taken together, these observations suggest that the susceptibility of different geographic populations to *H. pylori* strains and *H. pylori*-associated disease differed.

### Risk of gastric cancer and peptic ulcer disease development associated with *cagA* status and *vacA* s, m, and i genotypes

Of the 13 examined studies, 6 investigated the associations between gastroduodenal diseases and *vacA* s- and m-region genotypes [33,35-37,41,42], and 2 investigated disease associations based on the *vacA* i-region genotype [33,41].

**Figure 2:**
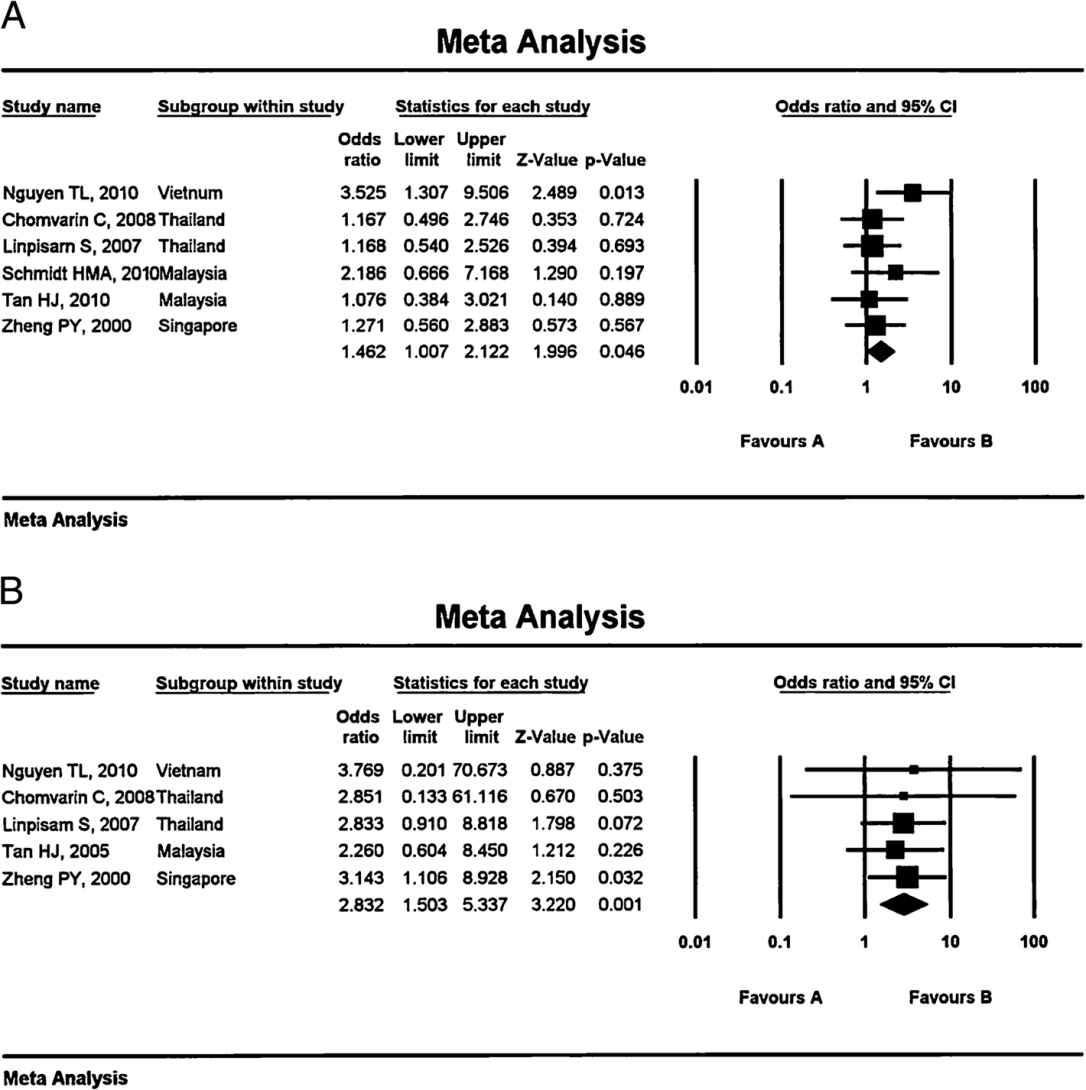
**Results of the meta-analysis for the risk of peptic ulcer disease in infections associated with *vacA *m1 genotype (A) and *cagA*-positive (B) *H. pylori *strains**

Nguyen et al. [33] reported that the frequency of *vacA* m1 alleles among *H.pylori* strains isolated from patients with peptic ulcer disease was 71% (17/24) in Vietnam, but only 41% with NUD (31/76; *p* = 0.146), although the difference was not significant. However, in combined analysis using Southeast Asian strains, the frequencies of *vacA* s1, m1, and i1 genotypes in *H. pylori*-infected NUD were 99%, 55%, and 94%, respectively, which were similar frequencies to those found in patients with peptic ulcer disease (100%, 59%, and 100%, respectively) and gastric cancer (100%, 54% and 100%, respectively) (Table 4). In meta-analysis, infection with *vacA* m1 type strains increased the risk of peptic ulcer development (OR: 1.46, 95%CI: 1.01-2.12, *p* = 0.046) in the Southeast Asian population (Figure 2A). However, the *vacA* s1 and i1 genotypes were not associated with an increased risk of either peptic ulcer disease or gastric cancer.

**Table 4:** **Summary of *cagA *and *vacA *s, m, and i genotypes in relation to peptic ulcer disease and gastric cancer risk**

				***cagA *genotype**		***vacA *genotype**												
**Disease**	**Paper (n)**	**Patient (n)**	***cagA *(n/%)**	**Western EPIYA-ABC**	**East-Asian EPIYA-ABD**	**s1 (n/%)**	**s1a (n/%)**	**s1b (n/%)**	**s1c (n/%)**	**s2 (n/%)**	**m1 (n/%)**	**m2 (n/%)**	**s1m1 (n/%)**	**s1m2 (n/%)**	**s2m1 (n/%)**	**s2m2 (n/%)**	**i1 (n/%)**	**i2 (n/%)**
NUD	8	604	555 (92)	127 (44)	160 (56)	414 (99)	62 (63)	4 (4)	33 (33)	4 (1)	224 (55)	185 (45)	224 (55)	180 (44)	0 (0)	4 (1)	186 (94)	11 (6)
PU	8	279	264 (95)	11* (21)	42 (79)	236 (100)	38 (64)	1 (2)	20 (33)	1 (0)	135 (59)	92 (41)	134 (59)	92 (41)	0 (0)	1 (0)	40 (100)	0 (0)
GC	5	74	73 (99)	9* (17)	45 (83)	42 (100)	2 (67)	0 (0)	1 (33)	0 (0)	22 (54)	19 (46)	22 (54)	19 (46)	0 (0)	0 (0)	22 (100)	0 (0)
**Total**		957	892 (93)	147 (37)	247 (63)	692 (99)	102 (63)	5 (3)	54 (34)	5 (1)	380 (56)	296 (44)	380 (56)	291 (43)	0 (0)	5 (1)	248 (96)	11 (4)

*: *p* < 0.05 (significantly different prevalence compared with NUD) by use of chi-square test.

Eight studies investigated the association between gastroduodenal disease and *cagA* status [32,33,35-37,41,42], while four reported the relationship between disease and *cagA* type [32,39-41]. In the meta-analysis, infection with *cagA*-positive strains increased the risk of peptic ulcer disease (OR: 2.83, 95%CI: 1.50-5.34, *p* = 0.001) in Southeast Asian populations. Schmidt et al. [40] reported that among Chinese patients with gastric cancer, the prevalence of EPIYA-ABD, -ABCC, and -ABC motif genotypes was 85.7%, 14.3%, and 0%, respectively. Moreover, these same authors later reported that the prevalence of the EPIYA-ABC and -ABD genotypes in Malaysia were 52.5% and 47.5% in patients with NUD, 13.6% and 86.4% with gastric cancer, and 31.3% and 68.7% with duodenal ulcer, respectively. The occurrence of East Asian-type strain (EPIYA-ABD) was higher in patients with peptic ulcer disease and gastric cancer than in those infected with Western-type strain (EPIYA-ABC) (Table 4). The prevalence of Western-type strain (EPIYA-ABC) in patients with NUD, peptic ulcer disease, and gastric cancer were 44% (127/287), 21% (11/53), and 17% (9/55), respectively, and 56%, 79%, and 83%, respectively, for East Asian-type strain (EPIYA-ABD). The risk of gastric cancer and peptic ulcer disease development in patients infected with East Asian-type strain (EPIYA-ABD) was significantly increased (OR: 3.03, 95%CI: 1.50-6.13, *p* < 0.002 and OR: 3.97, 95%CI: 1.87-8.43, *p* < 0.001, respectively).

## Discussion

Highly virulent genotypes of *H. pylori* are associated with gastric epithelial damage, including gastric mucosal atrophy, in infected patients [30]. Notably, the *vacA* s1, m1, and i1 genotypes and *cagA*-positive strains of *H. pylori* are linked to elevated inflammatory cell infiltration compared to that induced by *vacA* s2, m2, and i2 genotypes and *cagA*-negative strains [48,49]. In the present study, our meta-analyses revealed a significant relationship between *H. pylori* virulence factor-associated genotypes, particularly *vacA* m-region genotype and *cagA* status, and an increased risk for the development of peptic ulcer disease in Southeast Asian populations.

An important characteristic of Southeast Asian countries is that several geographic populations live together. Recently, Breurec et al. [50] reported that five major types of historical human migration patterns have occurred in Southeast Asia: i) migration from India introducing hpEurope bacteria into Thailand, Cambodia, and Malaysia; ii) migration of the ancestors of Austro-Asiatic speaking people carrying hspEAsia bacteria into Vietnam and Cambodia; and iii) migration of the ancestors of the Thai people from Southern China into Thailand carrying *H. pylori* of population hpAsia2; iv) migration of Chinese to Thailand and Malaysia within the last 200 years resulting in the spread of hspEAsia strains; and v) migration of Indians to Malaysia within the last 200 years distributing both hpAsia2 and hpEurope bacteria. Therefore, both Western and East Asian strains according to EPIYA motif genotyping can be observed in Southeast Asia. East Asian-type strain (EPIYA-ABD) was associated with an approximately three-fold increased risk of disease. Therefore, we concluded that the risk of developing *H. pylori*-associated diseases varies among different geographic populations despite living simultaneously in the same country.

The “Asian paradox” might be explained by the widespread prevalence of weakly cytotoxic strains and correspondingly low frequency of *H. pylori-*associated diseases. However, here, the prevalence of strains with the *vacA* s1 region among Southeast Asia populations was similar to that of East Asian and Latin American populations, who have a higher risk of gastric carcinogenesis [7,9,10]; half of the isolated *H. pylori* strains were Western-type strains with an EPIYA-ABC genotype. The sequence of the EPIYA-D type perfectly matches the high-affinity binding sequence for SHP2 domains of SHP-2 (pY-[V/T/A/I/S]-X-[L/I/V]-X-[F/W]) [51,52]. In contrast, the sequences of the EPIYA-C type differ from the SHP-2 binding sequence by a single amino acid located in the pY + 5th position [51,52]. As a result, East Asian-type CagA exhibits stronger binding activity for SHP-2 and a greater ability to induce morphological changes in epithelial cells than the Western type. The estimated age-standardized incidence rates of gastric cancer in Vietnamese patients (24.4 and 14.6/100,000 in men and women, respectively) infected with East Asian-strains of *H. pylori* are relatively higher than those in Thailand (4.2 and 3.0/100,000 in men and women, respectively) and Malaysia (10.7 and 6.4/100,000), where the majority of the population is Thai and Malay, and are infected with Western-type strains ( http://globocan.iarc.fr/). Therefore, the lower prevalence of higher virulence strains (e.g., ABD type) may explain the low frequency of gastric cancer observed in Southeast Asia, particularly in the southern part.

In Vietnam, *H. pylori* infection was detected at a rate of approximately 66% and was shown to be strongly associated with active gastritis, atrophy and intestinal metaplasia [33]. As genetic characteristics of *H. pylori* in Vietnam, the majority of isolates possess *cagA*, *oipA***“**on**”**, *vacA* s1, and *vacA* i1 genes, while the incidence of *vacA* m1 gene is less frequent [7,31,33]. Here, the prevalence of the *vacA* m1 genotype in patients residing in Hanoi and Ho Chi Minh were 58% and 34%, respectively. The prevalence of peptic ulcer disease in Hanoi, located in the northern part of Vietnam, is significantly higher than that in the southern city of Ho Chi Minh, despite similarities of ethnicity and diet [33]. Moreover, the age-standardized incidence rate of gastric cancer in Hanoi was approximately 1.5-fold higher than that in Ho Chi Minh (27.0 vs. 18.7 cases per 100,000 males, respectively) [33]. The higher prevalence of peptic ulcer disease and gastric cancer observed in Hanoi might be attributable to the higher prevalence of *H. pylori* strains carrying *vacA* m1 in this region. Although Vietnam is located between regions with a high and low risk of gastric cancer, the rate of gastric cancer in Vietnam is approximately three times lower than that in Japan and Korea, a finding that might also explain the low prevalence of *H. pylori* strains carrying *vacA* m1 (58% vs. nearly 100% in East Asia) [7,16,53].

Thailand has a population of 60 million people and is comprised of two major ethnic groups: Thai and Chinese. Eight types of *H. pylori* strains were identified in Thailand; half of the strains possessed genotypes typically found in South Asia (*cagA* type 2a/cag right-end junction type III and *vacA* s1a-m1c), while the other half consisted of genotypes typically encountered in East Asia (*cagA* type 1a/cag right-end junction type II and *vacA* s1c-m1b) based on combinations of *cagA* EPIYA motif genotype, genotype at the right end of the *cag* pathogenicity island into five subtypes according to deletion, insertion and substitution motifs (e.g., Type II strains were predominant in China and Japan and type III strains were most common in India and type III strains were typical of strains from India) and *vacA* s-region genotype [7,54]. Thailand was considered to be a cross roads with respect to *H. pylori* genotypes, as isolates from ethnic Thai were commonly either South/Central Asian or mixed genotypes (East Asian and South/Central Asian) based on the *cagA*, *cag*PAI, and *vacA* genes, whereas isolates from ethnic Chinese were typically the East-Asian genotype [55]. Although the incidence of gastric cancer in Thailand is lower than that of East-Asian populations, the incidence of gastric cancer is not rare among Chinese living in Thailand, as the majority of gastric cancers (82%) occurred among ethnic Chinese or Thai-Chinese. Therefore, the incidence of gastric cancer varies among different geographic populations, and the high prevalence of higher-virulence strains may be associated with the high frequency of gastric cancer in Chinese residing in Thailand, which is typically a low-incidence area.

In Malaysia, three distinct ethnic groups predominate: Malays, Chinese, and Indians. Chinese and Indians have migrated to Malaysia for nearly three consecutive generations. The *vacA* s1c genotype was also the predominant genotype detected among the Chinese patients residing in Malaysia, while s1a was predominant in Indians and Malays in Kuala Lumpur [37].

The present study has some limitations. First, in this study, because the prevalence of *cagA*-negative strains was very low in Southeast Asian countries, we did not demonstrate the relationship between risk of *H. pylori*-related diseases and *cagA*-negative strains, which are 20-80% of *H. pylori* strains isolated from US and European population. Second, there was no information about biochemical role among different *H. pylori* virulent factors (e.g., *cagA* status, *cagA* EPIYA motif and *vacA* genotypes) in pathogenesis for peptic ulcer disease and gastric cancer development [56]. Recently, Mueller, et al. [56] reported association of c-Src and c-Abl kinases and phosphorylation of EPIYA motif, and differences of phosphorylation between East Asian-type strain (EPIYA-ABD) and Western type strain (EPIYA-ABC). Therefore, further studies will be required to clarify the role of *H. pylori cagA* EPIYA motif and *vacA* genotype for the development of gastrointestinal diseases.

## Conclusion

We demonstrated that the prevalence of specific *vacA* m-region and *cagA* EPIYA motif genotypes was found to vary significantly among the respective Southeast Asian countries. Moreover, our present meta-analyses identified a significant relationship between *vacA* m-region genotype and *cagA* status and the development of diseases in Southeast Asian. Importantly, most of the *H. pylori* strains isolated from countries with high incidences of gastric cancer concurrently possessed virulent genotypes such as *vacA* s1/m1 and East Asian-type *cagA*[7]. In contrast, in countries with a low gastric cancer incidence, such as Thailand and Malaysia, a considerable proportion of *H. pylori* isolates exhibited less virulent genotypes, such as *vacA* m2 and Western-type *cagA*[7]. Based on the age-standardized incidence rate of gastric cancer, Asian countries can be categorized as either high-risk (e.g., Japan, Korea, and China), intermediate-risk (e.g., Vietnam), or low-risk (e.g., Thailand and Indonesia). The clinical usefulness of *cagA* EPIYA motif and *vacA* genotype testing must be evaluated in studies using a large number of individuals with *cagA* EPIYA motif and *vacA* genotypes.

## Abbreviations

CI: Confidence intervals; *H. pylori*: *Helicobacter pylori*; NUD: Non-ulcer dyspepsia; OR: Odds ratios; PCR: Polymerase chain reaction.

## Competing interests

The authors declare no competing interests related to this study.

## Author contributions

SS, MS, R-KV, VM, HM, TF, and YY designed and performed research; SS and MS analyzed data and wrote the paper. All authors read and approved the final manuscript.

## Disclosure

The authors declare that there are no conflicts of interest with regard to this work.

## Grant support

A grant-in-aid from the Ministry of Education, Culture, Sports, Science and Technology of Japan (23590913).

## Pre-publication history

The pre-publication history for this paper can be accessed here:

http://www.biomedcentral.com/1471-2334/12/223/prepub
